# Supplementary material for: A Tutorial Review of Bayesian Optimization with Gaussian Processes to Accelerate Stationary Point Searches
Source: ACS Phys Chem Au. 2026 May 20;6(4):633–64. doi: 10.1021/acsphyschemau.6c00038 (PMC13397454; doi:10.1021/acsphyschemau.6c00038)
Supplement: Supplementary file 1 [file pg6c00038_si_001.pdf]

A Tutorial Review of Bayesian Optimization with Gaussian Processes to Accelerate Stationary Point Searches

Rohit Goswami*

Institute IMX and Lab-COSMO, École polytechnique fédérale de Lausanne (EPFL), Station 12, CH-1015 Lausanne, Switzerland

E-mail: rgoswami@ieee.org

The Supporting Information collects algorithm flowcharts, marginal-likelihood landscapes, hyperparameter defaults, and implementation details.

S1 Algorithm Flowcharts

The following flowcharts visualize the classical and GP-accelerated versions of the dimer method and NEB. Each pair shows the unmodified algorithm (left) alongside its GP-accelerated counterpart (right).

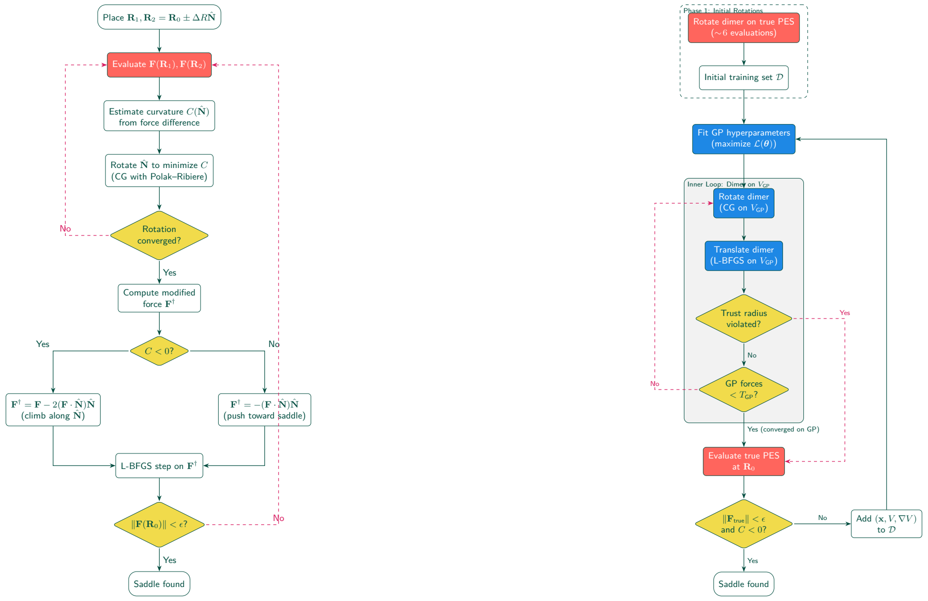

Figure S1: Classical dimer (left) and GP-dimer (right) flowcharts. The classical version evaluates the true PES at every rotation step; the GP-accelerated version performs rotations and translations on the surrogate surface, querying the oracle only when trust radius violations occur or GP-level convergence is achieved.

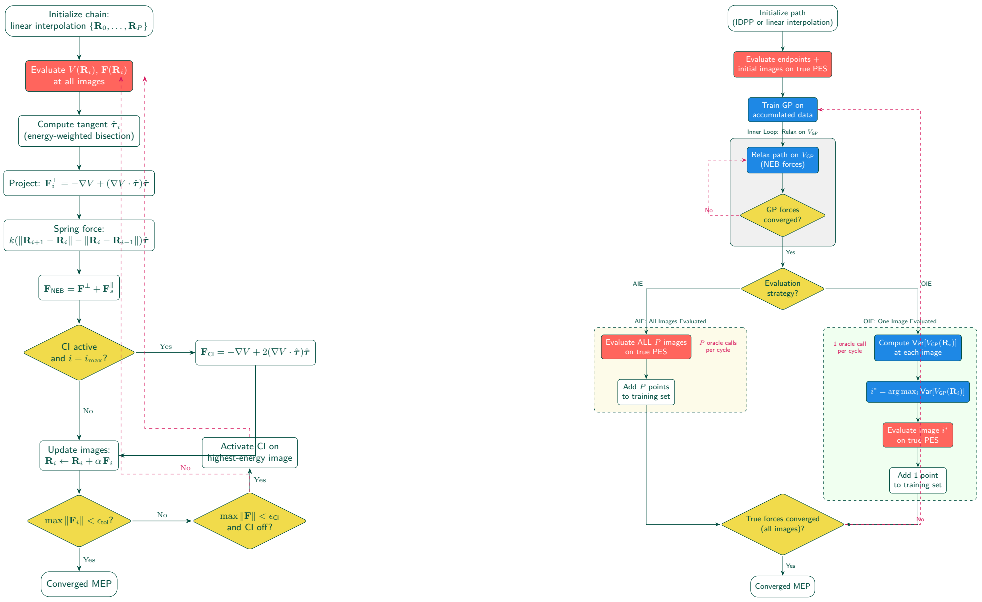

Figure S2: Classical NEB (left) and GP-NEB (right) flowcharts. The classical version optimizes all images via L-BFGS; the GP-NEB variant uses one-image evaluation with a configurable acquisition rule (pure variance or UCB in the current implementation) to reduce oracle queries.

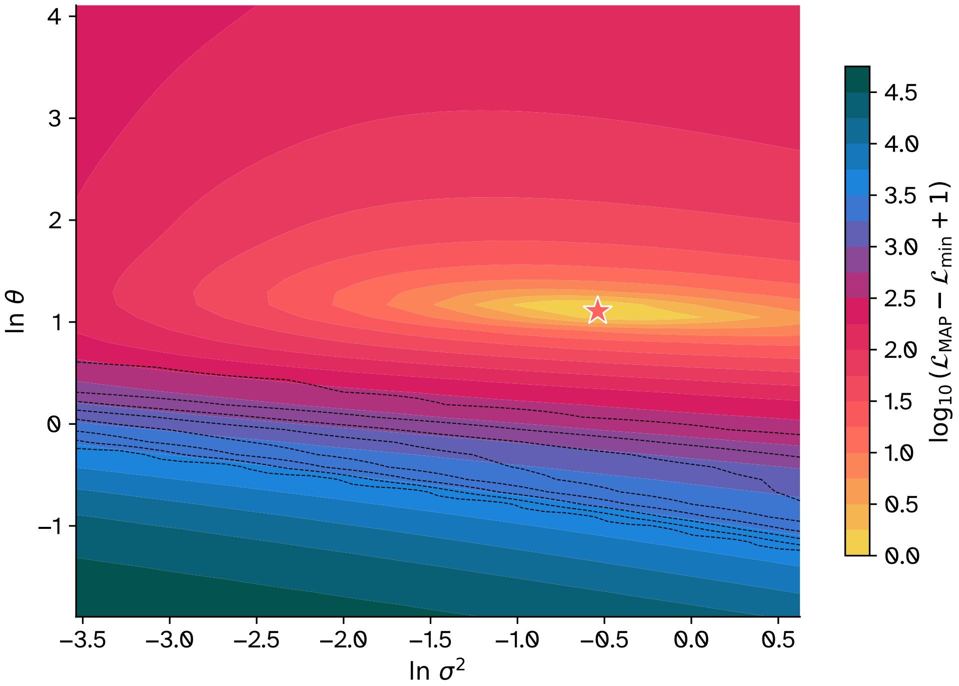

Figure S3: MAP-regularized negative log marginal likelihood landscape on the LEPS surface. The filled contours show the NLL as a function of the log-hyperparameters $\ln \sigma^2$ and $\ln \theta$, computed on a 40×40 grid from 5 training points near the reactant. The dashed black contours show the gradient norm. The coral star marks the MAP optimum that SCG converges to. Regions where the Cholesky factorization fails (hyperparameters that produce a non-positive-definite covariance matrix) are masked.

S2 Marginal Likelihood Landscape

S3 Hyperparameter Defaults

Table S1 collects default values for all hyperparameters used in GP-dimer, GP-NEB, and GP-minimization. These are starting values tuned for molecular systems with DFT energies (eV scale) and small to medium-sized systems (10-50 atoms). Adjustments may be needed for model potentials, very large systems, or different energy units.

Table S1: Default hyperparameters for GP-accelerated saddle point searches. Parameters marked with † are documented in.[1]

**Parameter**	**Default**	**Meaning**
Kernel		
σ_c^2	1.0	Constant offset (eV-scale energies); set to 0.0 for centered model surfaces
σ_f^2	Free (MLL)	Signal variance (optimized via MAP-NLL)
$l_{\phi(i,j)}$	Free (MLL)	Inverse-distance length scales (per atom-type pair, optimized)
Noise and Regularization		
σ_E^2	10^{-8}	Energy noise (Tikhonov regularizer, not physical noise)
σ_F^2	10^{-8}	Force noise (Tikhonov regularizer)
Jitter	$10^{-8}\max(\mathrm{diag}(\mathbf{K}))$	Initial Cholesky jitter; grows 10× per retry
MAP Regularization†		
μ_0	10^{-4}	Initial logarithmic barrier strength (Eq. 47)
α	10^{-3}	Barrier growth rate with training set size
$\mu_{\max}$	0.5	Maximum barrier strength
$\lambda_{\max}$	ln(2)	Upper bound for $\log \sigma_f^2$ (barrier ceiling)
Dimer Method		
ΔR	0.01 Å	Dimer midpoint-to-endpoint separation (`dimer_sep`, Eq. 6)
T_{GP}	10^{-3}	GP-level force convergence threshold (eV/Å)
FPS Subset Selection		
$M_{\mathrm{sub,init}}$	10	Initial FPS subset size
$M_{\mathrm{sub,max}}$	30	Maximum subset size (after oscillation retries)
Trust Radius		
$T_{\min}$	0.1 Å	Minimum trust radius (per-atom EMD)
$\Delta T_{\mathrm{explore}}$	0.4 Å	Exploration increment (saturation curve amplitude)
N_{half}	5	Half-life for exponential saturation (Eq. 49)
a_{floor}	0.3 Å	Minimum physical ceiling (small systems)
a_A	1.0 Å	Atomic length scale for size-dependent ceiling (Eq. 50)
NEB Acquisition		
κ_{UCB}	2.0	Exploration weight for Upper Confidence Bound (OIE)
Random Fourier Features		
D_{RFF}	200	Number of random features (for large training sets $M > 100$)

S4 Connection to Code: chemgp-core

The chemgp-core crate (source at `https://github.com/lode-org/ChemGP`, documentation at `https://lode-org.github.io/ChemGP/`) is the pedagogical reference implementation of the algorithms described in this review. Each listing below is extracted from the crate source, and the same binary runs the illustrative benchmarks reported throughout. Production-scale studies use the C++ gpr\$_{optim}$ code reported in the companion JCTC and ChemPhysChem papers;[1,2] chemgp-core shares the same algorithmic core and was aligned with gpr\$_{optim}$ through a ten-fix campaign covering rotation fitting, convergence criteria, trust radius, and rigid-body projection. Table S2 summarizes the module correspondence.

Table S2: Conceptual mapping between mathematical ideas, chemgp-core Rust modules, and gpr_optim C++ classes. Both implementations share the same algorithmic structure; the Rust code runs the benchmarks reported in this review.

Concept	chemgp-core (Rust)	gpr_optim (C++)
Inverse-distance kernel	`kernel.rs` (`MolInvDistSE`)	`InvDistSE`
Derivative kernel blocks	`kernel.rs` (`molinvdist_kernel_blocks`)	`GPKernelBlocks`
GP training (SCG)	`train.rs` + `scg.rs` + `nll.rs`	`GPModel`
Dimer method	`dimer.rs` (`gp_dimer`)	`DimerSearch`
NEB method	`neb.rs` + `neb_oie.rs`	`NEBSearch`
OT-GP Dimer	`otgpd.rs` (`otgpd`)	`OTGPDimer`
FPS + EMD	`sampling.rs` + `emd.rs`	`FPSSampler`, `EMDDistance`
Trust regions	`trust.rs`	`TrustRegion`
Random Fourier features	`rff.rs` (`build_rff`)	`RFFModel`
L-BFGS	`lbfgs.rs` + `optim_step.rs`	`LBFGS`
Constant kernel	`covariance.rs` + `rff.rs`	–

Table S3: Equation-to-function mapping for chemgp-core. Each equation in the main text maps to a specific function in the Rust implementation. The annotations in the code listings connect variable names to the mathematical symbols in the equations.

Equation	chemgp-core Function	Purpose
Eq. 7	`curvature()`	Dimer curvature estimate
Eq. 8	`rotational_force()`	CG rotation force
Eq. 9	`translational_force()`	Householder reflection
Eq. 11	`neb_force()`	NEB total force
Eq. 23–24	`molinvdist_kernel_blocks()`	Derivative covariance blocks
Eq. 25	`build_full_covariance()`	Assemble $\mathbf{K}_{\text{full}}$
Eq. 22	`robust_cholesky()`	Guarded Cholesky factorization
Eq. 20	`predict()`	Posterior mean prediction
Eq. 21	`predict_with_variance()`	Posterior mean + variance
Eq. 31	`invdist_jacobian()`	Inverse-distance Jacobian
Eq. 45–46	`emd_distance()`	EMD distance (per-type + overall)
Eq. 50	`adaptive_trust_threshold()`	Physical trust ceiling
Eq. 51	`build_rff()`	RFF model construction

The code snippets below are extracted from chemgp-core, with annotations connecting variables to the equations in the preceding sections. The same functions run the examples in this review and are deployed via the eOn saddle point search framework[3] for production calculations.

S4.1 Kernel Evaluation and Analytical Derivative Blocks

The kernel evaluation (Eq. 28) computes the squared Mahalanobis distance in inverse-distance feature space and applies the SE exponential. The variable `d2` accumulates the sum $\sum_{(i,j)} \theta^2_{(i,j)}(\phi_{ij}(\mathbf{x}) - \phi_{ij}(\mathbf{x}'))^2$, where `inv_lengthscales` stores the $\theta_{(i,j)} = 1/l_{\phi(i,j)}$ values and `compute_inverse_distances` returns the feature vector $\boldsymbol{\phi}(\mathbf{x})$.

Parameterization convention: The paper's kernel (Eq. 28) uses the standard SE form with a 1/2 factor in the exponent: $k(\mathbf{x}, \mathbf{x}') = \sigma^2 \exp(-1/2 \sum_i (\phi_i(\mathbf{x}) - \phi_i(\mathbf{x}'))^2 / l_i^2)$. The code absorbs the 1/2 into the inverse lengthscale definition, so $\theta_{\text{code}} = 1/(l_{\text{paper}}\sqrt{2})$. The two forms are mathematically equivalent; the code's parameterization simplifies the derivative computation.

```
impl MolInvDistSE {
    pub fn eval(&self, x: &[f64], y: &[f64]) -> f64 {
```

```
        let fx = compute_inverse_distances(x, &self.frozen_coords
            );
        let fy = compute_inverse_distances(y, &self.frozen_coords
            );
        let mut d2 = 0.0;
        if !self.feature_params_map.is_empty() {
            for i in 0..fx.len() {
                let idx = self.feature_params_map[i];
                let val = (fx[i] - fy[i]) * self.inv_lengthscales
                    [idx];
                d2 += val * val;
            }
        } else {
            let theta = self.inv_lengthscales[0];
            for i in 0..fx.len() {
                let diff = fx[i] - fy[i];
                d2 += diff * diff;
            }
            d2 *= theta * theta;
        }
        self.signal_variance * (-d2).exp()
    }
}
```

The derivative blocks (`kernel_blocks`, Listing S1 in the Supporting Information) compute the four covariance components between energy and force observations via chain rule through the inverse-distance Jacobian.

S4.2 Covariance Matrix Assembly and GP Training

The full covariance matrix (Eq. 25) is assembled by calling `kernel_blocks` for every pair of training configurations and placing the resulting 2×2 block structure at the

appropriate indices. For N training points with $D = 3N_{\text{atoms}}$ coordinates each, the matrix has dimension $N(1+D) \times N(1+D)$: the first N rows/columns correspond to energies, and the remaining ND to forces. The noise variances σ_E^2 and σ_F^2 are added to the respective diagonal blocks.

```
pub fn build_full_covariance(
    kernel: &Kernel, x_data: &[f64], dim: usize, n: usize,
    noise_e: f64, noise_g: f64, jitter: f64, const_sigma2: f64,
) -> Mat<f64> {
    let total = n * (1 + dim);
    let mut k_mat = Mat::<f64>::zeros(total, total);
    for i in 0..n {
        let xi = &x_data[i * dim..(i + 1) * dim];
        let b = kernel.kernel_blocks(xi, xi);
        k_mat[(i, i)] = b.k_ee + const_sigma2 + noise_e + jitter;
            // const_sigma2: added to all E-E entries (rank-1
          matrix sigma_c^2 * 11^T)
        let s_g = n + i * dim;
        for d in 0..dim {
            k_mat[(i, s_g + d)] = b.k_ef[d];
            k_mat[(s_g + d, i)] = b.k_fe[d];
            k_mat[(s_g + d, s_g + d)] += noise_g + jitter;
        }
        for di in 0..dim {
            for dj in 0..dim {
                k_mat[(s_g + di, s_g + dj)] = b.k_ff[(di, dj)];
            }
        }
        for j in (i + 1)..n {
            let xj = &x_data[j * dim..(j + 1) * dim];
            let b = kernel.kernel_blocks(xi, xj);
            let j_s = n + j * dim;
```

```
            k_mat[(i, j)] = b.k_ee + const_sigma2;
            k_mat[(j, i)] = b.k_ee + const_sigma2;
            for d in 0..dim {
                k_mat[(i, j_s + d)] = b.k_ef[d];
                k_mat[(j_s + d, i)] = b.k_ef[d];
                k_mat[(s_g + d, j)] = b.k_fe[d];
                k_mat[(j, s_g + d)] = b.k_fe[d];
            }
            for di in 0..dim {
                for dj in 0..dim {
                    k_mat[(s_g + di, j_s + dj)] = b.k_ff[(di, dj)
                       ];
                    k_mat[(j_s + dj, s_g + di)] = b.k_ff[(di, dj)
                       ];
                }
            }
        }
    }
    // Floor sub-epsilon entries (matches MATLAB GPstuff: C(C<eps
       )=0)
    let eps = f64::EPSILON;
    for r in 0..total {
        for c in 0..total {
            if k_mat[(r, c)].abs() < eps { k_mat[(r, c)] = 0.0; }
        }
    }
    k_mat
}
```

The training loop (`train_model`, Listing S2) minimizes the MAP-regularized NLL (Eq. 32) using the SCG optimizer[4] with log-space reparameterization.

The GP-dimer main loop (`gp_dimer`, Listing S3) alternates between outer iterations

(oracle evaluations that grow the training set) and inner iterations (rotation and translation on the GP surface). Convergence requires both small translational force and negative curvature. The trust radius check (Eq. 42 or Eq. 48) breaks the inner loop when exceeded.

The GP-NEB OIE outer loop (`gp_neb_oie`, Listing S4) selects images via configurable acquisition strategies: `MaxVariance`, `Ucb` (NEB force plus perpendicular uncertainty; the default), or `ExpectedImprovement`. A critical implementation detail: acquisition uses forces computed *before* inner relaxation, because re-predicting at relaxed positions with sparse training data produces unreliable NEB forces.

The RFF model (`build_rff`, Listing S5) replaces the exact GP with Bayesian linear regression in a random feature space sampled from the kernel's spectral density (Section 8.4 of the main text). Prediction reduces to a dot product at $\mathcal{O}(D_{\text{rff}})$ cost per component. Activating RFF is a single configuration change:

```
let config = NEBConfig {
    rff_features: 500,       // 0 = exact GP; >0 = RFF
       approximation
    max_gp_points: 40,       // per-bead subset for hyperparameter
        training
    acquisition: AcquisitionStrategy::Ucb,
    lcb_kappa: 2.0,          // UCB exploration weight
    const_sigma2: 0.0,       // constant kernel: 1.0 for molecular
        PES, 0.0 for models
    ..Default::default()
};
```

S4.3 Code Listings

The listings here cover the inverse-distance kernel derivative blocks (Listing S1), MAP-regularized hyperparameter training (Listing S2), GP-dimer main loop (Listing S3), GP-NEB OIE acquisition loop (Listing S4), and Random Fourier feature construction (Listing S5).

*Listing S1.

S5 Derivative blocks for the inverse-distance kernel (kernel_blocks).

The chain-rule structure projects the feature-space Hessian to Cartesian coordinates via the inverse-distance Jacobians.

```
pub fn molinvdist_kernel_blocks(
    k: &MolInvDistSE, x1: &[f64], x2: &[f64],
) -> KernelBlocks {
    let (f1, j1) = invdist_jacobian(x1, &k.frozen_coords);
    let (f2, j2) = invdist_jacobian(x2, &k.frozen_coords);
    let nf = f1.len();

    // Per-feature theta^2 values
    let theta2: Vec<f64> = (0..nf).map(|i| {
        let idx = if k.feature_params_map.is_empty() { 0 }
                  else { k.feature_params_map[i] };
        k.inv_lengthscales[idx].powi(2)
    }).collect();

    // SE kernel value
    let r: Vec<f64> = (0..nf).map(|i| f1[i] - f2[i]).collect();
    let d2: f64 = (0..nf).map(|i| theta2[i] * r[i] * r[i]).sum();
    let kval = k.signal_variance * (-d2).exp();



    // Feature-space gradient: dk/df
    let mut dk_df2 = vec![0.0; nf];
    let mut dk_df1 = vec![0.0; nf];
```

```
    for i in 0..nf {
        let v = 2.0 * kval * theta2[i] * r[i];
        dk_df2[i] = v;
        dk_df1[i] = -v;
    }
    // Feature-space Hessian: H[i,j] = 2*kval*(theta2[i]*delta_ij
        - 2*u[i]*u[j])
    let u: Vec<f64> = (0..nf).map(|i| theta2[i] * r[i]).collect()
      ;
    let mut h_feat = Mat::<f64>::zeros(nf, nf);
    for i in 0..nf {
        h_feat[(i, i)] = 2.0 * kval * (theta2[i] - 2.0 * u[i] * u
            [i]);
        for j in (i + 1)..nf {
            let val = -4.0 * kval * u[i] * u[j];
            h_feat[(i, j)] = val;
            h_feat[(j, i)] = val;
        }
    }

    // Chain rule: project to Cartesian coordinates
    let k_ee = kval;
    let k_ef = mat_t_vec(&j2, &dk_df2);  // J2^T * dk/df2  (D x
      1)
    let k_fe = mat_t_vec(&j1, &dk_df1);  // J1^T * dk/df1  (D x
      1)
    let k_ff = jt_h_j(&j1, &h_feat, &j2); // J1^T H J2     (D x D
      )
    KernelBlocks { k_ee, k_ef, k_fe, k_ff }
}
```

*Listing S2.

S6 MAP-regularized hyperparameter training via SCG (train_model).

All hyperparameters are optimized in log-space; Cholesky failure returns infinity to reject infeasible configurations.

```
pub fn train_model(model: &mut GPModel, iterations: usize) {
    // Pack hyperparameters to log-space
    let mut w0 = Vec::with_capacity(1 + model.kernel.n_ls_params
        ());
    w0.push(model.kernel.signal_variance().ln());
    for &l in model.kernel.inv_lengthscales() {
        w0.push(l.ln());
    }
    let w_prior = w0.clone();
    let prior_var = compute_prior_variances(&model.kernel);

    let mut fg = |w: &[f64]| -> (f64, Vec<f64>) {
        nll_and_grad(w, &model.x_data, model.dim, model.n_train,
                     &model.y, &model.kernel, model.noise_var,
                     model.grad_noise_var, model.jitter,
                     &w_prior, &prior_var, model.const_sigma2,
                     model.prior_dof, model.prior_s2, model.
                        prior_mu)
    };

    let config = ScgConfig {
        max_iter: iterations,
        tol_f: 1e-4,
        lambda_init: model.scg_lambda_init,
        ..Default::default()
    };
```

```
    let result = scg_optimize(&mut fg, &w0, &config);
    if result.converged || result.f_best < f64::INFINITY {
        let sigma2 = result.w_best[0].exp();
        let inv_ls: Vec<f64> =
            result.w_best[1..].iter().map(|v| v.exp()).collect();
        model.kernel = model.kernel.with_params(sigma2, inv_ls);
    }
}
```

*Listing S3.

S7 GP-dimer main loop (gp_dimer).

The outer loop evaluates the oracle and grows the training set; the inner loop performs rotation and translation on the GP surface.

```
pub fn gp_dimer(
    oracle: &OracleFn, x_init: &[f64], orient_init: &[f64],
    kernel: &Kernel, config: &DimerConfig,
) -> DimerResult {
    let mut state = DimerState {
        r: x_init.to_vec(),
        orient: normalize_vec(orient_init),
        dimer_sep: config.dimer_sep,
    };
    let mut td = TrainingData::new(x_init.len());

    // Bootstrap: evaluate midpoint and one dimer endpoint
    let (e, g) = oracle(x_init);
    td.add_point(x_init, e, &g);
    let r1 = dimer_endpoint(&state);
```

```
let (e1, g1) = oracle(&r1);
td.add_point(&r1, e1, &g1);

for _outer in 0..config.max_outer_iter {
    // FPS subset selection for hyperparameter training
    let td_sub = select_fps_subset(&td, &state.r, config);

    // Train GP on subset, predict on full data (RFF if
       configured)
    let mut gp = GPModel::new(kernel.clone(), &td_sub, ...);
    train_model(&mut gp, config.gp_train_iter);
    let model = build_pred_model(&gp.kernel, &td, config);

    // Inner loop: rotate + translate on GP surface
    for _inner in 0..config.max_inner_iter {
        rotate_dimer(&mut state, &model, config);
        let (g0, g1, _e0) = predict_dimer_gradients(&state, &
           model);
        let f_trans = translational_force(&g0, &state.orient)
           ;
        if vec_norm(&f_trans) < config.t_force_gp { break; }
        let r_new = translate_dimer_lbfgs(&state, &g0, &g1,
           config);
        if exceeds_trust(&r_new, &td, config) { break; }
        state.r = r_new;
    }

    // Evaluate oracle at proposed position
    let (e_true, g_true) = oracle(&state.r);
    td.add_point(&state.r, e_true, &g_true);
```

```
        // Converge when true force is small AND curvature is
           negative
        let f_true = translational_force(&g_true, &state.orient);
        if vec_norm(&f_true) < config.t_force_true && curvature <
            0.0 {
            return DimerResult { converged: true, oracle_calls:
               td.npoints(), .. };
        }
    }
    DimerResult { converged: false, oracle_calls: td.npoints(),
       .. }
}
```

*Listing S4.

S8 GP-NEB OIE acquisition loop (gp_neb_oie).

The select_image function supports MaxVariance, Ucb, and ExpectedImprovement strategies.

```
pub fn gp_neb_oie(
    oracle: &OracleFn, x_start: &[f64], x_end: &[f64],
    kernel: &Kernel, config: &NEBConfig,
) -> NEBResult {
    let mut images = init_path(x_start, x_end, config);
    let mut td = TrainingData::new(x_start.len());

    // Evaluate endpoints and midpoint
    for x in &[x_start, x_end, &images[config.images / 2]] {
        let (e, g) = oracle(x);
        td.add_point(x, e, &g);
    }
```

```
    for _outer in 0..config.max_outer_iter {
        let model = train_and_build_model(&td, kernel, config);

        // Acquire: select unevaluated image by acquisition
           strategy
        let i_eval = select_image(
            &config.acquisition, &images, &energies,
            &unevaluated, &model, &cached_forces, config,
        );

        // Evaluate oracle at selected image
        let (e, g) = oracle(&images[i_eval]);
        td.add_point(&images[i_eval], e, &g);

        // Relax path on GP surface (L-BFGS inner loop)
        images = oie_inner_relax(&model, &images, &td, config);

        // Convergence check on true forces
        let max_f = compute_neb_forces(&images, &model, config).
           max_f;
        if max_f < config.conv_tol { /* verify unevaluated images
            */ }
    }
}
```

*Listing S5.

S9 Random Fourier feature construction (`build_rff`).

Frequency vectors are sampled from the kernel's spectral density (Bochner's theorem); the constant kernel adds one extra basis function.

```
pub fn build_rff(
    kernel: &Kernel, x_train: &[f64], y_train: &[f64],
    dim: usize, n: usize, d_rff: usize,
    noise_var: f64, grad_noise_var: f64, seed: u64,
    const_sigma2: f64,
) -> RffModel {
    let inv_ls = kernel.inv_lengthscales();
    let d_feat = kernel.n_features(dim);

    // Sample frequencies from N(0, 2*theta^2 * I) (Bochner's
       theorem)
    let mut rng = StdRng::seed_from_u64(seed);
    let mut w = Mat::<f64>::zeros(d_rff, d_feat);
    for f in 0..d_feat {
        let idx = kernel.pair_type_index(f);
        let scale = (2.0f64).sqrt() * inv_ls[idx];
        for i in 0..d_rff {
            w[(i, f)] = rng.sample::<f64, _>(StandardNormal) *
               scale;
        }
    }
    let b: Vec<f64> = (0..d_rff).map(|_| rng.random::<f64>() *
       2.0 * PI).collect();
    let c = kernel.signal_variance().sqrt() * (2.0 / d_rff as f64
       ).sqrt();

    // Design matrix Z: d_eff = d_rff + 1 (extra column for const
        kernel)
    let d_eff = d_rff + 1;
    let n_obs = n * (1 + dim);
    let mut z = Mat::<f64>::zeros(n_obs, d_eff);
```

```
    for i in 0..n {
        let (zi, j_z) = rff_features(&w, &b, c, &x_train[i*dim..(
            i+1)*dim]);
        for f in 0..d_eff { z[(i, f)] = zi[f]; }          // Eq.
            rff_features
        for d in 0..dim {
            for f in 0..d_eff { z[(n + i*dim + d, f)] = j_z[(f, d
                )]; }
        }
    }

    // Bayesian linear regression: A = Z^T diag(prec) Z + I
    let prec = build_precision(n, dim, noise_var, grad_noise_var)
        ;
    let a = zt_diag_z_plus_eye(&z, &prec, d_eff);
    let llt = a.llt(Side::Lower).expect("RFF␣Cholesky␣failed");
    let rhs = zt_diag_y(&z, &prec, y_train, d_eff);
    let alpha = llt.solve(&rhs);
    RffModel { w, b, c, alpha, a_chol: llt, dim, const_sigma2, ..
        }
}
```

S10 Mathematical Derivations

S10.1 Rigid-Body Mode Basis Construction

The projection of rigid-body modes requires an orthonormal basis $\{\mathbf{u}_k\}_{k=1}^{6}$ spanning the 6 external degrees of freedom. For a molecule with N atoms and Cartesian coordinates $\mathbf{x} = (x_1, y_1, z_1, \ldots, x_N, y_N, z_N)^T \in \mathbb{R}^{3N}$, the six basis vectors correspond to three translations and three infinitesimal rotations.

The unnormalized translation vectors are:

$$\mathbf{t}_x = (1,0,0,1,0,0,\dots,1,0,0)^T, \tag{S1}$$

$$\mathbf{t}_y = (0,1,0,0,1,0,\dots,0,1,0)^T, \tag{S2}$$

$$\mathbf{t}_z = (0,0,1,0,0,1,\dots,0,0,1)^T. \tag{S3}$$

The unnormalized infinitesimal rotation vectors (about the molecular center of mass $\mathbf{r}_0$) are:

$$\mathbf{r}_x = (0, z_1 - z_0, -(y_1 - y_0), \dots, 0, z_N - z_0, -(y_N - y_0))^T, \tag{S4}$$

$$\mathbf{r}_y = (-(z_1 - z_0), 0, x_1 - x_0, \dots, -(z_N - z_0), 0, x_N - x_0)^T, \tag{S5}$$

$$\mathbf{r}_z = (y_1 - y_0, -(x_1 - x_0), 0, \dots, y_N - y_0, -(x_N - x_0), 0)^T. \tag{S6}$$

where (x_i, y_i, z_i) are the coordinates of atom i, and (x_0, y_0, z_0) is the center of mass.

These six vectors are linearly independent but not orthonormal. The Gram-Schmidt process applied in the order $(\mathbf{t}_x, \mathbf{t}_y, \mathbf{t}_z, \mathbf{r}_x, \mathbf{r}_y, \mathbf{r}_z)$ produces the orthonormal basis $\{\mathbf{u}_k\}_{k=1}^{6}$:

$$\mathbf{u}_1 = \frac{\mathbf{t}_x}{\|\mathbf{t}_x\|}, \tag{S7}$$

$$\mathbf{u}_2 = \frac{\mathbf{t}_y - (\mathbf{t}_y \cdot \mathbf{u}_1)\mathbf{u}_1}{\|\mathbf{t}_y - (\mathbf{t}_y \cdot \mathbf{u}_1)\mathbf{u}_1\|}, \tag{S8}$$

$$\mathbf{u}_3 = \frac{\mathbf{t}_z - \sum_{j=1}^{2}(\mathbf{t}_z \cdot \mathbf{u}_j)\mathbf{u}_j}{\|\mathbf{t}_z - \sum_{j=1}^{2}(\mathbf{t}_z \cdot \mathbf{u}_j)\mathbf{u}_j\|}, \tag{S9}$$

$$\mathbf{u}_{k+3} = \frac{\mathbf{r}_k - \sum_{j=1}^{k+2}(\mathbf{r}_k \cdot \mathbf{u}_j)\mathbf{u}_j}{\|\mathbf{r}_k - \sum_{j=1}^{k+2}(\mathbf{r}_k \cdot \mathbf{u}_j)\mathbf{u}_j\|}, \quad k \in \{x, y, z\}. \tag{S10}$$

In practice, the translation vectors are already orthogonal (and of equal norm $\sqrt{N}$), so the Gram-Schmidt process only needs to orthogonalize the rotation vectors against the translations and against each other. This basis is computed once per molecular geometry and cached; the projection then costs $\mathcal{O}(N)$ per step.

S10.2 Hyperparameter Oscillation Detection

The oscillation diagnostic mentioned in Section 8.2 of the main text detects when the MAP estimate of hyperparameters oscillates between competing local minima as new data arrives. For a hyperparameter vector $\boldsymbol{\theta}(t) = (\theta_1(t), \ldots, \theta_K(t))$ at outer iteration t, the per-component oscillation indicator is:

$$O_j(t) = \begin{cases} 1, & \text{if } (\theta_j(t) - \theta_j(t-1))(\theta_j(t-1) - \theta_j(t-2)) < 0, \\ 0, & \text{otherwise.} \end{cases} \tag{S11}$$

This indicator is 1 when hyperparameter j reverses direction (sign change in the gradient) between consecutive steps, and 0 otherwise. The fraction of oscillating components over a sliding window of length W (typically $W = 5$ in chemgp-core) is:

$$f_{\text{osc}}(t) = \frac{1}{KW} \sum_{j=1}^{K} \sum_{s=t-W+1}^{t} O_j(s). \tag{S12}$$

When $f_{\text{osc}}(t)$ exceeds a threshold p_{osc} (default $p_{\text{osc}} = 0.8$ in chemgp-core), the algorithm detects instability and triggers growth of the FPS subset M_{sub} (Section 8.1). The subset grows incrementally (default: +2 points per retry) up to a maximum size (default: 30). This adaptive subset sizing sharpens the MLL landscape by adding geometrically diverse training data, constraining the optimizer to a narrower region of hyperparameter space.

S10.3 Kernel Block Structure

The full covariance matrix for derivative observations has a block structure that arises from the chain rule applied to the inverse-distance feature map. Throughout this section and in `chemgp-core/src/kernel.rs`, these derivative blocks are written for energy gradients ∇V; atomic forces enter later through the separate convention $\mathbf{F} = -\nabla V$. For two configurations $\mathbf{x}_1$ and $\mathbf{x}_2$, the kernel blocks are:

$$k_{ee} = k(\phi(\mathbf{x}_1), \phi(\mathbf{x}_2)) \tag{S13}$$

$$k_{ef} = \frac{\partial k}{\partial \mathbf{x}_2} = \left(\frac{\partial k}{\partial \boldsymbol{\phi}}\right)^T \frac{\partial \boldsymbol{\phi}}{\partial \mathbf{x}_2} = \mathbf{J}_2^T \frac{\partial k}{\partial \boldsymbol{\phi}} \tag{S14}$$

$$k_{fe} = \frac{\partial k}{\partial \mathbf{x}_1} = \mathbf{J}_1^T \frac{\partial k}{\partial \boldsymbol{\phi}} \tag{S15}$$

$$k_{ff} = \frac{\partial^2 k}{\partial \mathbf{x}_1 \partial \mathbf{x}_2^T} = \mathbf{J}_1^T \left(\frac{\partial^2 k}{\partial \boldsymbol{\phi} \partial \boldsymbol{\phi}^T} - 2 \frac{\partial k}{\partial \boldsymbol{\phi}} \otimes \frac{\partial \boldsymbol{\phi}}{\partial \mathbf{x}_2} \right) \mathbf{J}_2 \tag{S16}$$

where $\mathbf{J}_i = \partial \boldsymbol{\phi} / \partial \mathbf{x}_i$ is the Jacobian of the feature map at configuration $\mathbf{x}_i$, and the feature-space Hessian is computed analytically for the SE kernel.

S10.4 Earth Mover's Distance for Trust Regions

The EMD-based trust region uses the per-type distance (Eq. 45) to compute a size-independent metric. For two configurations $\mathbf{x}_1$ and $\mathbf{x}_2$ with atom types $\{t_i\}$, the per-type distance is:

$$d_{\text{EMD}}^{(t)} = \min_{\pi \in \Pi_t} \sum_{i \in \mathcal{I}_t^{(1)}} \sum_{j \in \mathcal{I}_t^{(2)}} c_{ij}^{(t)} \pi_{ij} \tag{S17}$$

where Π_t is the set of joint distributions with marginals matching the counts of type t atoms, and $c_{ij}^{(t)} = \|\mathbf{x}_1^{(i)} - \mathbf{x}_2^{(j)}\|$ is the ground cost. To match the implementation in `chemgp-core/src/emd.rs`, the per-type transport cost is converted to a mean displacement by dividing by the number of atoms of that type, and the overall intensive EMD is the maximum over atom types:

$$d_{\text{EMD}}(\mathbf{x}_1, \mathbf{x}_2) = \max_t \frac{d_{\text{EMD}}^{(t)}}{N_t} \tag{S18}$$

This is the same convention as Eq. 46 in the main text.

S10.5 OIE Acquisition Criterion Derivation

The Upper Confidence Bound (UCB) acquisition criterion for NEB OIE balances exploitation (large NEB forces) against exploration (high uncertainty). The criterion is:

$$\alpha(\mathbf{R}_i) = |\mathbf{F}_i^{\text{NEB}}| + \kappa \cdot \sigma_\perp(\mathbf{R}_i, \boldsymbol{\tau}_i) \tag{S19}$$

where $\sigma_\perp$ is the perpendicular gradient variance (Eq. 36). This is derived from the Gaussian tail bound: with probability at least $1 - \delta$,

$$|F_d^{\text{true}} - F_d^{\text{GP}}| \leq \kappa \cdot \sigma(F_d^{\text{GP}}), \quad \kappa = \sqrt{2\log(1/\delta)} \tag{S20}$$

Applying this bound to the NEB force magnitude gives the UCB criterion as a conservative estimate of the true force magnitude. Within Eq. S19, $\kappa = 0$ gives force-only selection. The separate pure-variance selector used by `AcquisitionStrategy::MaxVariance` is instead Eq. 38 from the main text, where image choice is based only on the GP energy variance.

S11 Benchmark Summary

Table S4 consolidates the oracle-call counts that appear across the figures of the main text and the runs shipped with `chemgp-core`. The numbers come directly from the JSONL outputs of the corresponding example binaries (`cargo run --release --example <name>`) and use the convergence thresholds documented in those examples. For CI-based path-search methods, the convergence basis is the climbing-image force rather than the whole-band maximum force. Production-scale benchmarks across hundreds of molecular reactions are reported in the companion papers;[1,2] the table below reflects the illustrative cases used in this review and is intended for reproduction, not as a stand-alone validation. No repeated-run uncertainty intervals or wall-clock comparisons are claimed for this tutorial table; wall time is reported separately in the benchmark harness rather than in the main tutorial summary. The toy-surface rows are included as pedagogical controls and

negative cases, not as molecular validation of the inverse-distance kernel. In the executable examples these rows use the Cartesian toy-surface kernel path, whereas the molecular benchmarks use the inverse-distance kernel; the toy-surface call counts are therefore not evidence for or against the molecular speedups attributed to the inverse-distance representation.

Table S4: Oracle-call counts to convergence for the illustrative benchmarks used in this review. Each row corresponds to a `chemgp-core` example whose execution trace drives a figure in the main text or a literature-aligned molecular benchmark in the accompanying ChemGP harness. "Baseline" denotes the classical reference algorithm for that task. "Variant A" and "Variant B" are method-specific comparison columns so the table can mix minimization, dimer, and CI-targeted path-search examples without forcing them into one GP/OT-GP taxonomy. Smaller is better.

Task	System	Variants	Baseline	Variant A	Variant B
Minimization	LEPS (9D)	gp_minimize vs L-BFGS	57	9	-
Minimization	Muller-Brown	gp_minimize vs L-BFGS	34	50	-
Minimization	PET-MAD molecule	gp_minimize vs L-BFGS	45	13	-
Single saddle	Muller-Brown	gp_dimer vs std. dimer	6	7	-
Single saddle	LEPS (9D)	gp_dimer / OTGPD vs dimer	10	8	8
Single saddle	d000 metatomic molecular run	standard dimer vs OTGPD	50	-	13
Path (NEB)	LEPS (9D)	gp_neb AIE / OIE vs NEB	156	100	42
Path (CI-NEB)	system100 (PET-MAD)	literature baseline OIE vs CI-NEB	112	-	23

For minimization, the GP overhead does not pay off uniformly: on the cheap Muller-Brown surface, where each oracle call has trivial cost, the GP loop loses to L-BFGS (50 vs 34 oracle calls). That row should be read as a negative control for the surrogate loop on a cheap Cartesian toy surface, not as a statement about the molecular inverse-distance kernel. The picture flips as the oracle becomes expensive: the PET-MAD minimization case shows a roughly threefold reduction, and the dimer and CI-targeted path-search rows show the largest oracle savings because the surrogate guides the search through anisotropic regions where direct methods make many short steps. The final system100 row should be read with its method definition in mind: the OIE benchmark is a CI-targeted comparison using the climbing-image force as the stopping basis ($CI|F| < 0.1$ eV/Å), not a whole-band max-force comparison. This pattern is consistent with the conditions under which surrogate acceleration is expected to help (Section 1, main text) and with the broader benchmarks in.[1,2]

S12 References

References

(1) Goswami, R.; Jónsson, H. Adaptive Pruning for Increased Robustness and Reduced Computational Overhead in Gaussian Process Accelerated Saddle Point Searches. *ChemPhysChem* **2026**, *27*, e202500730.

(2) Goswami, R.; Masterov, M.; Kamath, S.; Pena-Torres, A.; Jónsson, H. Efficient Implementation of Gaussian Process Regression Accelerated Saddle Point Searches with Application to Molecular Reactions. *Journal of Chemical Theory and Computation* **2025**, *21*, 7935–7943.

(3) Chill, S. T.; Welborn, M.; Terrell, R.; Zhang, L.; Berthet, J.-C.; Pedersen, A.; Jónsson, H.; Henkelman, G. EON: Software for Long Time Simulations of Atomic Scale Systems. *Modelling and Simulation in Materials Science and Engineering* **2014**, *22*, 055002.

(4) Møller, M. F. A Scaled Conjugate Gradient Algorithm for Fast Supervised Learning. *Neural Networks* **1993**, *6*, 525–533.
